# Albumin-glutaraldehyde glue for repair of superficial lung defect: an in vitro experiment

**DOI:** 10.1186/s13019-016-0443-x

**Published:** 2016-04-12

**Authors:** Maximilian Bures, Hans-Klaus Höffler, Godehard Friedel, Thomas Kyriss, Enole Boedeker, Florian Länger, Patrick Zardo, Ruoyu Zhang

**Affiliations:** Department of Cardiac, Thoracic, Transplantation and Vascular Surgery, Hannover Medical School, Hannover, Germany; Department of Thoracic Surgery, Center for Pneumology and Thoracic Surgery, Schillerhoehe Hospital, Teaching hospital of the University of Tuebingen, Solitudestr. 18, Gerlingen, Germany; Department of Pathology, Hannover Medical School, Hannover, Germany; Department of Cardiac and Thoracic Surgery, Otto-von-Guericke University Magdeburg, Magdeburg, Germany

**Keywords:** Lung, Air leak, Sealant, BioGlue

## Abstract

**Background:**

Albumin-glutaraldehyde glue gained a widespread acceptance in repair of superficial lung defects associated with alveolar air leaks (AAL). As its sealing efficacy has not yet been thoroughly corroborated by clinical studies, we sought to assess the properties of commercially available albumin-glutaraldehyde glue (BioGlue™) in an in vitro lung model.

**Methods:**

The lower lobe of freshly excised swine lung (*n* = 10) was intubated and ventilated. A focal superficial parenchymal defect (40 × 25 mm) was created on the inflated lung. AAL was assessed with increasing inspired tidal volume (TVi). After glue application, AAL was assessed until burst failure occurred. To evaluate glue elasticity, the length of defect was recorded in the inflated lung.

**Results:**

Superficial parenchymal defects resulted in AAL increasing with ascending TVi. Multiple linear regression analysis revealed strong correlation between AAL and maximal inspiratory pressure. There was one application error. At TVi = 400, 500, 600, 700, 800 and 900 ml, BioGlue™ achieved complete sealing in nine, six, five, four two and one specimens, respectively. Mean burst pressure was 38.0 ± 4.2 cmH_2_O. All sealant failures were cohesive. BioGlue™ allowed an expansion of covered lung defects of 1.5 ± 1.7 mm.

**Conclusions:**

Our in vitro tests demonstrated a high sealing efficacy of BioGlue™ for repair of superficial lung defects. Due to the rigid nature, caution should be taken to use this kind of sealant in trapped lungs.

## Abstrakt

***Hintergrund*:**

Die Wirksamkeit von chirurgischen Klebstoffen zur Behandlung der alveolo-pleuralem Luftleck (AAL) ist immer noch kontrovers diskutiert. Wir evaluierten in dieser Studie die Abdichtungswirksamkeit von einem Albumin-Glutaraldehyd-Kleber BioGlue™ mittels eines in vitro Lungenmodels.

***Methode*:**

Der Unterlappen von frisch entnommenen Schweinlungen (*n* = 10) wurde intubiert und beatmet. Eine pleurale Läsion (40 × 25 mm) wurde erstellt und APL mit steigendem inspiratorischem Tidalvolumen (TVi) untersucht. Nach Applikation von BioGlue™ wurde AAL auf die gleiche Weise gemessen bis zur Auftritt von Kleberbruch. Zur Untersuchung der Elastizität des Klebers wurde die Länge der pleuralen Läsion gemessen.

***Ergebnis*:**

Die oberflächliche pleurale Läsion führte bei aufsteigendem TVi zum Anstieg von AAL. Multiple lineare Regressionsanalyse ergab eine starke Korrelation zwischen AAL und dem maximalen inspiratorischen Druck. Applikationsfehler trat bei einem Test auf. Bei TVi = 400, 500, 600, 700, 800 und 900 ml führte BioGlue™ zur kompletten Versieglung in neun, sechs, fünf, vier, zwei und einem Test. Der mittlere Bruchdruck betrug 38.0 ± 4.2 cmH_2_O. Es wurde nur kohäsiver Kleberbruch gefunden. Nach der Kleberapplikation hat sich die Länge der pleuralen Läsion um 1.5 ± 1.7 mm gedehnt.

***Schlussfolgerung*:**

Unsere in vitro Versuche zeigten eine zuverlässige Versiegelung der oberflächlichen Lungendefekte durch BioGlue™. Aufgrund der Streifigkeit des Klebers ist Vorsicht bei der Verwendung derartigen Klebers an gefesselter Lunge geboten.

## Background

Superficial lung defect is a common intraoperative complication of lung surgery, particularly following pleural decortication, dissection of firm pleural adhesions and division of incomplete fissures [1–3]. It results in alveolar air leaks (AAL) associated with delayed removal of chest tubes, prolonged hospital stays, increased postoperative morbidity and patient discomfort [4, 5]. In the past decade surgical sealants have been increasingly used in treating AAL as adjuncts to conventional closing techniques including suturing and stapling. The commonly used sealants are human thrombin-fibrinogen sponge (TachoSil®; Takeda Pharmaceutical Company Limited, Osaka, Japan), albumin-glutaraldehyde glue (BioGlue™; CryoLife Europa Ltd., Surrey, UK) and synthetic polymer sealants (TissuePatch™, Tissuemed Ltd, Leeds, UK; CoSeal®, Baxter Healthcare, Fremont, CA) [5, 6]. However, a body of clinical trials assessing sealant efficacy yielded inconsistent results, thus raising questions about evidence-based sealant use in lung surgery [5–7]. This might be attributed to a number of factors like small sample sizes, inherent flaws in study design or heterogeneity of clinical characteristics in study and control groups [5, 8].

Bioglue™ (CryoLife Europa Ltd., Surrey, UK) is a two-component sealant composed of bovine serum albumin and glutaraldehyde [9]. The aldehyde groups of glutaraldehyde react with the amine groups of bovine serum albumin and those present in the extracellular matrix and cell surface, resulting in strong cross-linking after 2–3 min. Bioglue™ has been widely used in cardiovascular surgery due to its high hemostatic efficacy [10–12]. In recent years BioGlue™ gained widespread acceptance as an adjunct in treating AAL in lung surgery [13]. So far, the pneumostatic efficacy of BioGlue™ has not been thoroughly evaluated. In the present study we sought to examine sealing efficacy and elasticity of BioGlue™ for repair of superficial lung defects by means of an established in vitro lung model [8].

## Methods

### Experimental protocol

**Fig. 1:**
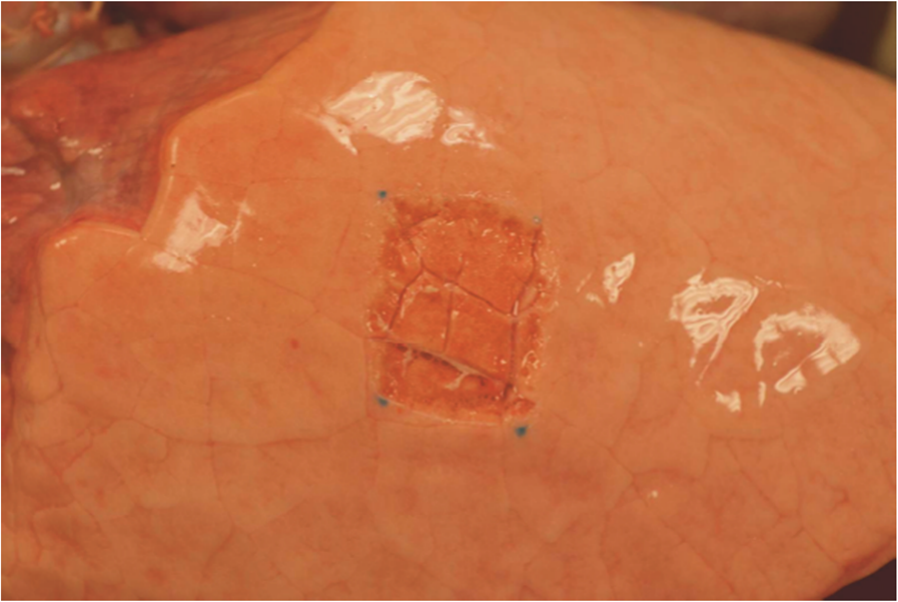
A superficial lesion (40 × 25 mm) on lower lobe with marker spots at cranial and caudal edge

Lungs of German Landrace pigs were freshly excised in a local slaughterhouse. Within two hours after harvest, the lungs were dissected along the trachea until the tracheal bifurcation was reached. The lower lobe was selectively intubated, ventilated and immersed in water to ensure impermeability. After being connected to the ventilation machine (Evita, Dräger, Lübeck, Germany), the lower lobe was ventilated in volume-controlled mode with a PEEP of 5 cmH_2_O, an I:E ratio of 1:2 and a frequency of 12/min. The lower lobe was fully inflated when inspiratory tidal volume (TVi) was ≥ 400 ml. Over-inflation of the lobe was observed with TVi ≥ 800 ml. To create a standardized superficial parenchymal lesion, a rectangle measuring 40 × 25 mm was marked on the fully inflated lower lobe with a marker pen. Using a small, conic headed drill, a defect was created by carefully applying pressure to the marked site, starting at the edges and advancing toward the middle of the designated area. Marker spots were then added to the cranial and caudal edge of the lesion (Fig. 1). TVi was increased in steps, recording respiratory parameters including expiratory tidal volume (TVe), maximal inspiratory pressure (Pmax), plateau pressure (Pplat), resistance and compliance for each subsequent step until a Pmax of 40 cmH_2_O was reached. AAL was calculated as difference between TVi and TVe.

**Fig. 2:**
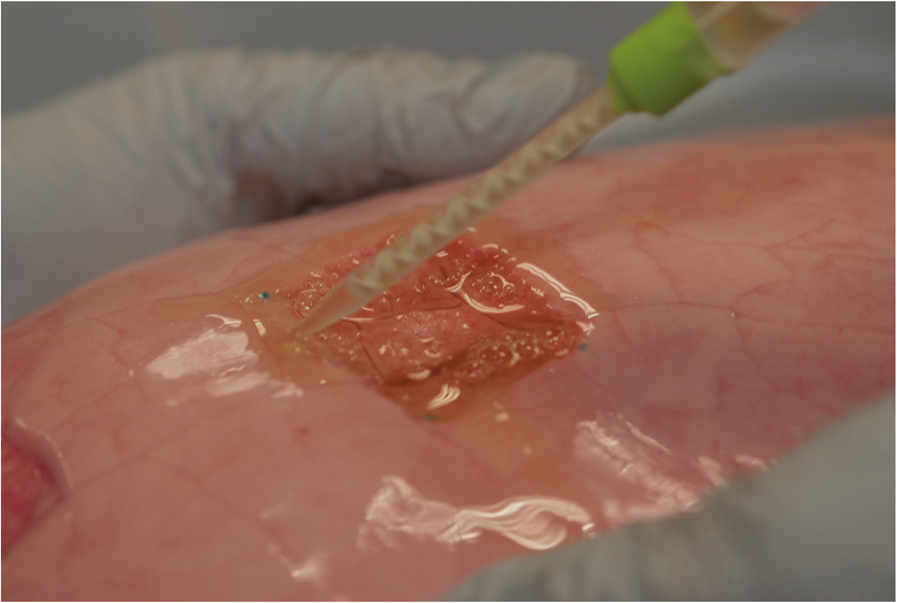
Application of BioGlue™ to the superficial lesion of lung parenchyma

Thereafter, BioGlue™ was applied to the lesion by carefully meandering along the surface, in compliance with user guidelines, and respecting a safety margin of one cm to all sides (Fig. 2). In the present experiment BioGlue™ was applied exclusively in two mL pre-filled syringes, using only one sample for each lesion. After 60 s full sealant adhesion was achieved and the glue hardened. The lower lobe was then ventilated again with TVi rising slowly from 100 ml. Commencing at 400 ml TVi the same ventilation parameters as before were recorded. AAL at the site of pleural defect were assessed by submersion tests and graded according to Macchiarini scale as grade 0 (no leak), grade 1 (countable bubbles), grade 2 (stream of bubbles) and grade 3 (coalescent bubbles) [14]. Sealing was considered successful, if no bubbles were observed (Macchiarini grade 0). The distance between both marker spots was measured to evaluate sealant elasticity. In case of sealant failure Pmax was recorded as burst pressure (BP). Pmax tolerated at the TVi, at which sealing was last achieved, was considered maximally tolerated pressure (MTP). Burst failure was furthermore categorized into adhesive or cohesive failure. Adhesive failure occurred at the interface between sealant and parenchymal defect. Cohesive failure was defined as failure within the sealant. Application error occurred, when cohesive or adhesive failure was observed before starting the test at 400 ml TVi. Two independent investigators noted all results. A third investigator would arbitrate any disagreement.

Finally, lung specimen containing the parenchymal lesion along with attached sealant were resected and fixed with glutaraldehyde. The specimen were embedded in paraffin and processed to obtain sections for haematoxylin-eosin staining.

### Statistical analysis

Normality of variables was tested using the Kolmogorov-Smirnov one-sample-test. Descriptive statistics are presented as mean ± standard deviation in case of normal distribution. Multiple linear regression was used to determine the ventilation parameters’ correlation with AAL. Statistical significance was assumed if *p* < 0.05. All statistical analysis was performed using SPSS (version 16.0 for Windows; SPSS Inc., Chicago, Illinois, USA).

## Results

A total of five pilot tests were performed prior to starting the experiment to ensure proper application of sealant and assessment of sealing efficacy. The results of these tests were not included in the data set or statistical analysis. Thereafter ten consecutive tests were undertaken. In the assessment before sealant application AAL increased with ascending TVi. Multiple linear regression analysis revealed strong correlation between AAL and Pmax (*p* < 0.001).

**Fig. 3:**
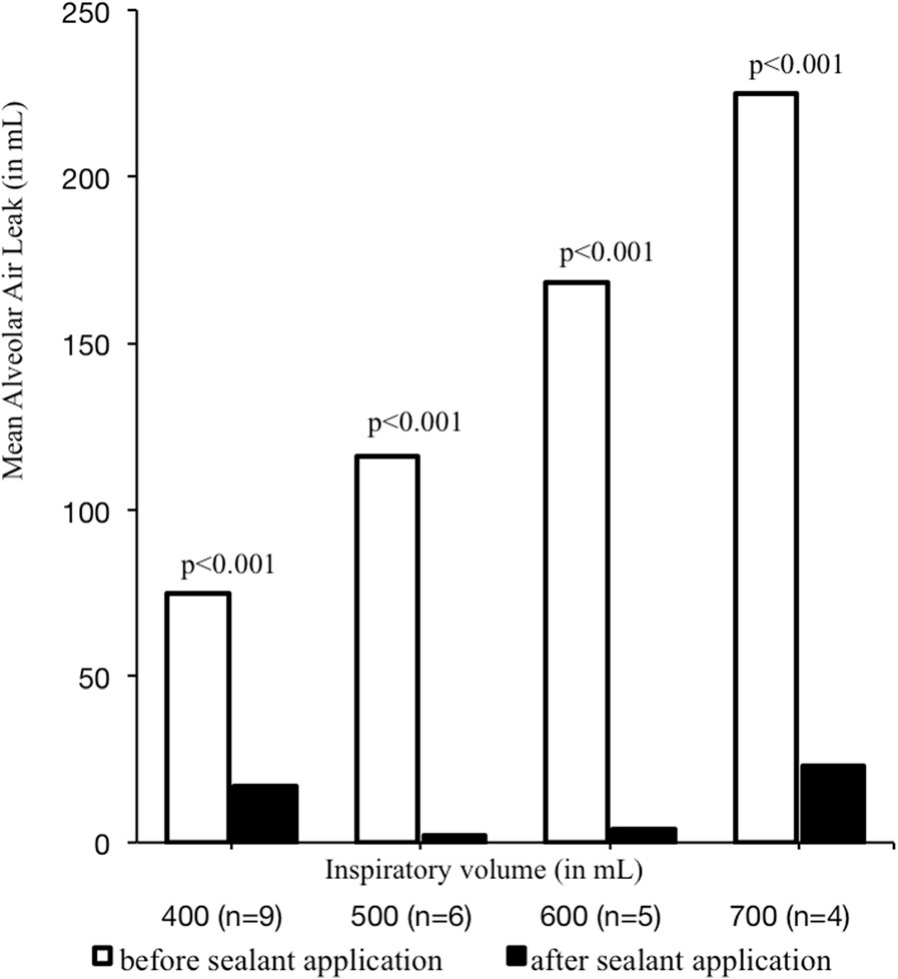
Graphic assessment of mean alveolar air leak before and after sealant application

We observed one application error. Following sealant application, nine lungs were still considered airtight at TVi of 400 ml, six at TVi of 500 ml, seven at TVi of 600 ml and four at TVi of 700 ml. Two lungs remained sealed even at TVi of 800 ml, with one lung remaining sealed until 1000 mL TVi. Graphic interpretation of AAL with ascending TVi is presented in Fig. 3. Mean BP was 38.0 ± 4.2 cmH_2_O and mean MTP was 36.3 ± 6.1 cmH_2_O. All burst failures were cohesive. BioGlue™ allowed an average length expansion of 1.5 ± 1.7 mm in relation to length at TVi of 400 ml.

**Fig. 4:**
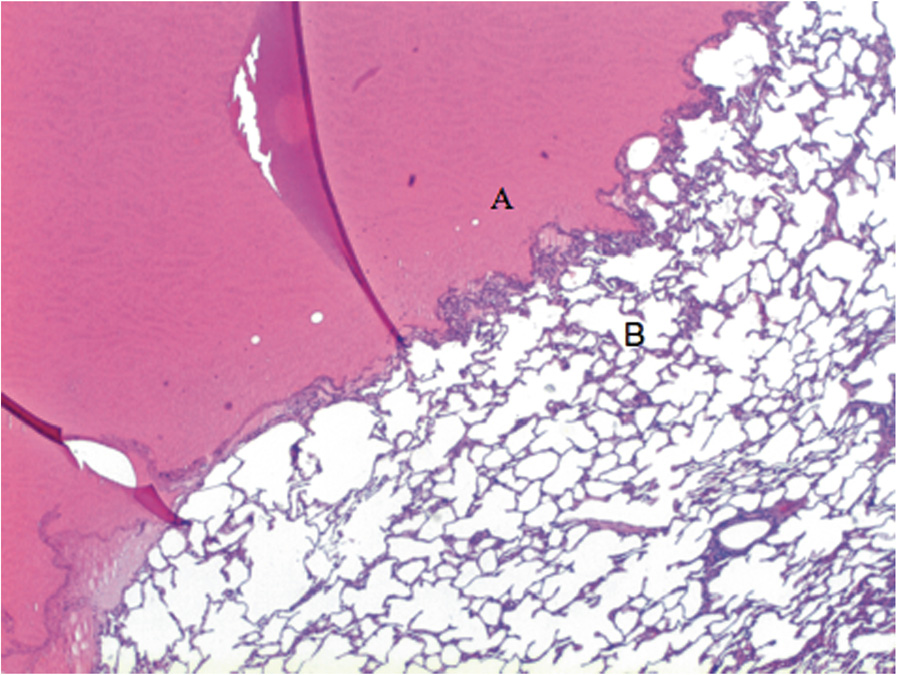
BioGlue™ glue (**a**) attached densely the underlying lung parenchymal lesion (**b**)

**Fig. 5:**
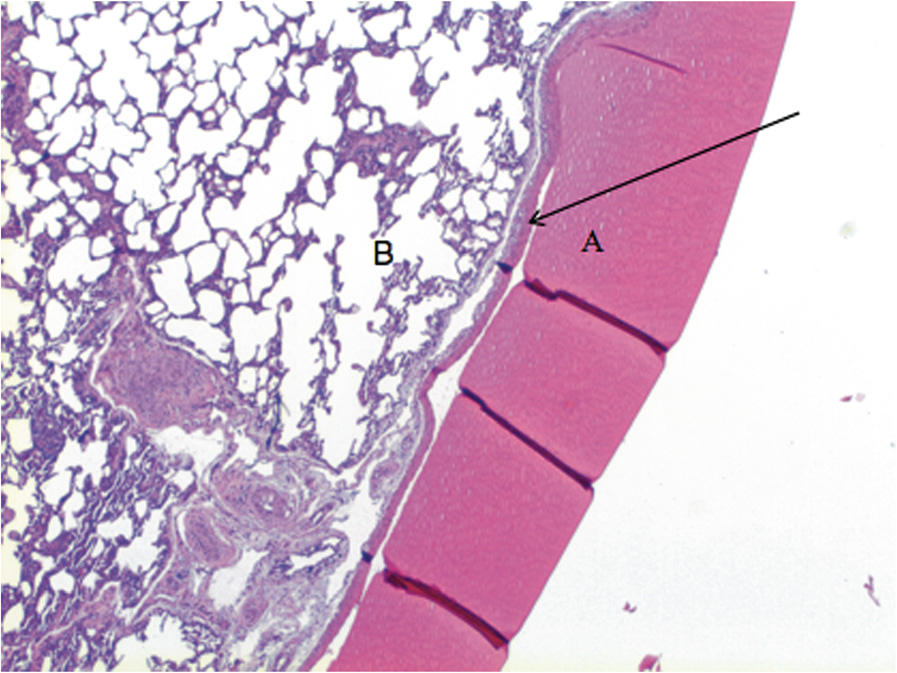
BioGlue™ glue (**a**) adhesive to the visceral pleura (*arrow*) with underlying parenchyma (**b**)

Haematoxylin-eosin staining of sealed lung specimen showed a sealant layer attaching densely to underlying parenchymal lesion (Fig. 4) and visceral pleura at the lesion edge (Fig. 5).

## Dicsussion

Superficial lesions of lung parenchyma occur regularly in lung surgery, especially in pleural decortation, dissection of firm pleural adhesions and division of incomplete fissures. One of the consequences is AAL resulting in delayed removal of chest tubes, prolonged hospital stays as well as higher postoperative morbidity [1, 3, 15]. The utility of surgical sealants as adjunct to conventional sealing techniques has been progressively recognized in lung surgery over the past decade [16, 17].

BioGlue™ is a two-component sealant consisting of bovine serum albumin and glutaraldehyde, and has been widely used for achieving haemostasis in cardiovascular surgery. In the recent past BioGlue™ has also seen an increase in use for repair of superficial lung defects. However, its true sealing efficacy remains unclear, which is at least partly due to inconsistent results of clinical trials. In a prospective, randomized controlled trial, Tansley et al. examined 52 patients with post-thoracotomy AAL and found significant differences in duration of intercostal drainage for patients treated with BioGlue™ over those in whom only surgical closing techniques were used [18]. However, this advantage could not be confirmed in another multicenter prospective randomized clinical trial, in which Allen and co-workers randomized 161 patients in a 2:1 ratio to receive topical application of BioGlue™ or control for at least one significant AAL following lung resection [19]. They found no significant difference in the duration to chest tube removal.

The present in vitro experiment aimed to test the sealing efficacy of BioGlue™ using an established lung model, which simulated a real scenario in lung surgery [8]. The results showed a high efficacy of BioGlue™ in treating AAL. BioGlue™ withstood very high inspiratory pressures, as reflected by the mean BP of 38.0 cmH_2_O – a respiratory pressure higher than in routine clinical settings. This suggests that BioGlue™ might be a useful tool for treatment of AAL, particularly in patients requiring aggressive mechanical ventilation. Albeit, the overall clinical benefits of BioGlue™ need to be further evaluated by well-designed prospective, randomized clinical trials. The authors are going to launch an experiment to compare BioGlue™ with other commonly used surgical sealants regarding sealing efficacy by means of the present in vitro lung model.

Additionally, our experiment analysed elastic properties of BioGlue™, which seem to comprise this sealant’s main weakness. After application of BioGlue™ only very little elasticity remained in the affected lung tissue, which might be of concern in patients with already trapped lung. Beside local effects, sealant run-off during application and ensuing restriction after glue hardening can further augment its rigidity. It appears that strong cross-links between albumin and glutaraldehyde, which ensure the great durability of BioGlue™, result in marked glue rigidity. This characteristic was previously mentioned by Belcher et al., where the sealant is described as having “a rigid, inelastic nature that does not expand with the underlying lung”, possibly increasing the risk for inflammation [20]. A study by Azadani et al. on mechanical properties of surgical glues revealed that BioGlue™ had a far lower compliance than comparable products [21]. Our tests provided quantitative data in this regard and could contribute to a deeper understanding of biomechanical properties of BioGlue™.

Concerning the bovine serum contained in Bioglue™, the risk of blood borne diseases exists for treated patients. Other reservations about the sealant concern toxicity of the glutaraldehyde component, as topified by Fürst et al. in a combined in vitro/in vivo experiment [22]. Here a cytotoxic effect of glutaraldehyde was found in vitro for human and mouse growth cells and an inflammation of the liver and aorta in vivo, suggesting BioGlue™ be used with caution.

Possible limitations of this in vitro trial include a certain inevitable variation in parenchymal lesion size. We minimized these potential confounders by using lungs from pigs of equal weight (around 80 kg) and size and by inducing standardized lesions. As all lower lobes were fully inflated with a TVi of 400 ml, no differences were noted in this regard. Judgment of sealing and measurement of lesion length might be prone to information bias. To counteract this, measurements were undertaken by two independent investigators and arbitrated by a third, if necessary. In the end our in vitro tests deliver quantitative results that may contribute to further understanding and use of BioGlue™ as adjunct therapy for AAL.

## Conclusions

Our in vitro tests suggest that BioGlue™ might be a useful adjunct in treating AAL, especially in patients requiring high ventilation pressures. Caution should be advised for the utility of this sealant in patients with trapped lung due to its rigidity.

## Abbreviations

AAL: alveolar air leak
MTP: maximally tolerated pressure
PEEP: positive end-expiratory pressure
Pmax: maximal inspiratory pressure
Pmean: mean inspiratory pressure
Pplat: plateau inspiratory pressure
TVe: expiratory tidal volume
TVi: inspired tidal volume.
